# Comparison of Severe COVID-19 Outcomes in Vaccinated and Unvaccinated Patients, with and Without Diabetes Mellitus in a Romanian Tertiary Healthcare Pneumology Hospital—A Retrospective Study

**DOI:** 10.3390/ijms27042082

**Published:** 2026-02-23

**Authors:** Ioana-Mădălina Moşteanu, Adela Gabriela Ştefan, Beatrice Mahler, Adina Mitrea, Ionela Mihaela Vladu, Oana-Andreea Parliţeanu, Diana Clenciu, Eugen Moţa, Maria Magdalena Roşu, Delia-Viola Reurean Pintilei, Beatrice Elena Vladu, Alexandru Stoichiță, Diana Cristina Protasiewicz-Timofticiuc, Theodora Claudia Radu-Gheonea, Ion-Cristian Efrem, Anca Maria Amzolini, Maria Moţa

**Affiliations:** 1 Doctoral School, University of Medicine and Pharmacy of Craiova, 200349 Craiova, Romania; madalina.mosteanu@yahoo.com (I.-M.M.); eugenmota@yahoo.com (E.M.); mmota53@yahoo.com (M.M.); 2 Institute of Pneumophtisiology “Marius Nasta”, 050159 Bucharest, Romania; oana_andreea@yahoo.com (O.-A.P.); alexandru.stoichita@drd.umfcd.ro (A.S.); 3 Department of Diabetes, Nutrition and Metabolic Diseases, Calafat Municipal Hospital, 205200 Calafat, Romania; adela.firanescu@yahoo.com; 4 Pneumology II Department, Faculty of Medicine, “Carol Davila” University of Medicine and Pharmacy, 050474 Bucharest, Romania; 5 Department of Diabetes, Nutrition and Metabolic Diseases, Faculty of Medicine, University of Medicine and Pharmacy of Craiova, 200349 Craiova, Romania; ionela.vladu@umfcv.ro (I.M.V.); diana.clenciu@umfcv.ro (D.C.); theodora.gheonea@umfcv.ro (T.C.R.-G.); 6 Department of Diabetes, Nutrition and Metabolic Diseases, Faculty of Midwives and Nursing, University of Medicine and Pharmacy of Craiova, 200349 Craiova, Romania; maria.rosu@umfcv.ro (M.M.R.); diana.protasiewicz@umfcv.ro (D.C.P.-T.); 7 Department of Medical-Surgical and Complementary Sciences, Faculty of Medicine and Biological Sciences, “Stefan cel Mare” University, 720229 Suceava, Romania; delia.pintilei@usm.ro; 8 Consultmed Medical Centre, Department of Diabetes, Nutrition and Metabolic Diseases, 700544 Iasi, Romania; 9 Faculty of Medicine, University of Medicine and Pharmacy of Craiova, 200349 Craiova, Romania; beatricevladu75@gmail.com; 10 Department of Internal Medicine—Medical Semiology, Faculty of Dentistry, University of Medicine and Pharmacy of Craiova, 200349 Craiova, Romania; cristian.efrem@umfcv.ro; 11 Department of Medical Semiology, Faculty of Medicine, University of Medicine and Pharmacy of Craiova, 200349 Craiova, Romania; anca.amzolini@umfcv.ro

**Keywords:** COVID-19, diabetes mellitus, inflammation, vaccination

## Abstract

The coronavirus disease 2019 (COVID-19) pandemic has had an unprecedented impact on public health. In the present study, we aimed to analyze the association of certain inflammatory biomarkers with severe COVID-19 and to explore the role of diabetes mellitus (DM) and vaccination status in relation to COVID-19 severity, intensive care need and mortality. Associated comorbidities (DM, obesity, cardiovascular, neurological, endocrine, hepatic, renal, pulmonary, rheumatological, psychiatric, hematological diseases, cancer and HIV), as well as inflammatory biomarkers, like ferritin, erythrocyte sedimentation rate (ESR), C-reactive protein (CRP), fibrinogen, lactate dehydrogenase (LDH), neutrophil-to-lymphocyte ratio (NLR), platelet-to-lymphocyte ratio (PLR), and systemic immune-inflammation index (SII) were analyzed in 866 subjects, according to vaccination status. In unvaccinated subjects, the highest AUROC curve for severe COVID-19 was recorded for CRP (0.668), and in the vaccinated group, the highest was recorded for SII (0.694). In age- and comorbidity-adjusted analyses, diabetes mellitus was associated with higher odds of severe COVID-19, ICU admission, and mortality among unvaccinated patients. This analysis was not feasible in the vaccinated group because of the very low number of unfavorable outcomes. These findings emphasize the potential role of vaccination in attenuating the excess risk linked to comorbidities—particularly diabetes mellitus—and support the use of accessible inflammatory biomarkers for early risk stratification. The results should be interpreted within the specific epidemiological phases of the pandemic and in the context of the observational study design.

## 1. Introduction

The coronavirus disease 2019 (COVID-19) pandemic has been associated with a high rate of infection, hospitalization, morbidity and mortality, and has had an unprecedented impact on public health [1,2]. The first cases of COVID-19 were reported in December 2019 in Wuhan, China, and the disease was declared a pandemic on 11 March 2020 [3,4].

In Romania, according to the National Institute of Public Health, the first confirmed case of COVID-19 was recorded on 26 February 2020, with approximately 95% of initially infected patients developing a mild form of the disease during hospitalization [5]. The situation worsened in May 2021, with the appearance of the fourth wave, due to the Delta variant of concern (VOC), with the first cases being reported in connection with Indian citizens [6]. The evolution of the Delta wave recorded an alarming upward trend until the end of October 2021, with approximately 1.6 million confirmed cases and almost 45,000 deaths by that date, putting an unimaginable pressure on the health system, both economically and socially [7]. A downward trend then followed, but on 4 December 2021, the first two cases infected with the Omicron VOC were confirmed, in two Romanian citizens with a travel history to South Africa [8]. With the start of the fifth wave in Romania, the situation escalated rapidly in the context of the winter holidays, due to the Omicron phenotype, characterized by increased transmissibility and risk of reinfection, while fortunately being associated with a reduced risk of severe disease and hospitalization [9,10]. Moreover, infection with Omicron VOC was associated with increased survival compared to Delta VOC [11]. Since the beginning of the pandemic and until 20 August 2023, a total of 3,413,851 confirmed cases of severe acute respiratory syndrome coronavirus 2 (SARS-CoV-2) infection have been reported, and a total of 68,266 deaths due to COVID-19. Over 90% of the deceased patients had at least one comorbidity, with cardiovascular diseases (71.6%) being the most common, followed by DM (29.6%), neurological diseases (21.2%), and obesity (18.5%) [12].

Thus, DM has been declared an independent risk factor for severe forms of COVID-19, as well as for a higher rate of hospitalizations and increased mortality [13,14,15]. DM influences the evolution of COVID-19 by altering the immune response due to the amplification of low-grade chronic systemic inflammation. This is characterized by reduced adiponectin levels and a consecutive blunted anti-inflammatory effect, as well as increased leptin secretion, with its pro-inflammatory role, among other pathophysiological effects [16,17]. The cytokine storm due to overproduction of pro-inflammatory cytokines affected the pulmonary alveoli, leading to severe acute respiratory syndrome, sepsis, and subsequently multiple organ dysfunction syndrome (MODS) [18]. In addition to immune dysfunction and chronic inflammation, hyperglycemia and related metabolic dysregulations have been acknowledged as having a pivotal role in the evolution of COVID-19 towards its most severe forms [19]. Moreover, conditions frequently coexisting with diabetes—such as obesity and obstructive sleep apnea (OSA)—further compound the risk. Obesity promotes a heightened inflammatory state and mechanical respiratory constraints, while OSA contributes to intermittent hypoxia and oxidative stress, collectively exacerbating COVID-19 severity in patients with diabetes [20].

Certain pro-inflammatory markers, such as interleukin-2 (IL-2), interleukin-6 (IL-6), interleukin-7 (IL-7), interleukin-10 (IL-10), and tumor necrosis factor (TNF), have been associated with an elevated risk of requiring intensive care support in patients with COVID-19 [18], while higher levels of IL-6, lactate dehydrogenase (LDH) and C-reactive protein (CRP) were correlated with respiratory failure [18,21]. Furthermore, increased IL-6 has been considered a predictor of unfavorable outcomes in patients with severe COVID-19 [18,22]. Inflammation-induced acute-phase reactants such as serum ferritin were similarly noted to predict disease severity, differentiating mild from severe COVID-19 [23,24]. Intensive care unit (ICU) admission and mortality were linked to neutrophil-to-lymphocyte ratio (NLR), while the platelet-to-lymphocyte ratio (PLR) was also predictive of disease severity. These two markers are simple and cost-effective methods that can be used for assessing inflammation [23].

The humoral and cellular immune responses are enhanced by vaccination, an essential step in preventing severe forms of COVID-19 [19,25]. However, despite the timely development of vaccines with increased efficacy in preventing both the development of COVID-19 and severe complications associated with the disease [26,27,28,29,30], SARS-CoV-2 infection remained a seasonal and endemic pathogen in the following years [19,31], with the disease being declared a stable health problem by the World Health Organization (WHO) on 5 May 2023 [32,33].

To date, limited data are available on the efficacy of COVID-19 vaccination among DM patients, its influence on associated comorbidities, symptom severity and immune response. In the present study, we aimed to analyze the association of certain inflammatory biomarkers with severe forms of COVID-19, as well as the contribution of DM to predicting COVID-19 severity, need for ICU admission and mortality, stratified by vaccination status.

## 2. Results

In this study, 866 hospitalized subjects with COVID-19 were evaluated for the presence or absence of DM and vaccination status. The results are presented within the context of two distinct pandemic phases corresponding to the availability of vaccination. A total of 910 subjects were selected for enrollment, but data were incomplete for 44 participants, so they were not included in the study. The statistical analysis included 866 eligible participants, of whom 87.8% were unvaccinated. This distribution reflects the temporal structure of the cohort, with unvaccinated patients predominantly enrolled during the 2020–2021 pre-vaccination period and vaccinated patients mainly admitted in 2022. The majority of unvaccinated subjects presented DM (64.2%), unlike the vaccinated subjects, among whom DM was found in only 35.8%. Among subjects with DM, most were treated with oral antidiabetic medication, while a smaller proportion received non-insulin injectable therapy or chronic insulin treatment prior to admission. During hospitalization, a substantial percentage required insulin therapy irrespective of previous regimen, reflecting the need for intensified glycemic management in the acute phase of COVID-19. The distribution of antidiabetic treatment was comparable between vaccinated and unvaccinated patients, supporting the assumption that baseline therapeutic patterns were similar across groups. The characteristics of the participants enrolled in this study regarding demographic data, DM treatment, vaccination status, clinical and biochemical data, and associated comorbidities are presented in Table 1.

While analyzing the vaccination status, a statistically significant difference was found between the unvaccinated and vaccinated subjects in terms of their environment, with the urban environment recording a higher percentage among the vaccinated group of participants compared to the unvaccinated group. A higher frequency of DM was recorded among unvaccinated vs. vaccinated subjects, with the percentage being almost double, as well as for other associated comorbidities, such as liver or kidney diseases. Also, a significantly higher frequency of severe forms of COVID-19 was observed among unvaccinated subjects, as well as a significantly higher frequency of intensive care unit (ICU) admission and mortality. Moreover, the duration of hospitalization was almost double, and the fasting plasma glucose (FPG) value was also higher in unvaccinated patients, compared to vaccinated ones, with the differences being significantly higher (*p* < 0.001).

Regarding the unvaccinated subjects, they were divided into two groups, DM (+) and DM (−). Participants with DM presented significantly higher FPG, as expected, and also a significantly increased percentage of associated comorbidities, with a high prevalence of cardiovascular diseases (88.5%), a twofold higher prevalence of obesity, and a higher frequency of liver, kidney and hematological diseases compared to DM (−) subjects. Patients with DM, compared to those without DM, presented a much longer duration of hospitalization (12 vs. 8 days), a significantly higher percentage of moderate and severe forms of COVID-19, as well as a much higher frequency of ICU admission (16.8% vs. 3.7%) and an alarming rate of mortality (14.5% vs. 0.4%) (*p* < 0.001).

Similarly, among vaccinated participants, DM (+) subjects showed a significantly increased FPG value, as well as a significantly higher percentage of associated diseases, such as cardiovascular (94.7%), liver (31.6%) and kidney (28.9%) diseases, as well as obesity (34.2%). Compared to DM (−) subjects, a 4-fold higher percentage of DM (+) patients were incompletely vaccinated (18.4% vs. 4.4%, *p* = 0.030). An extremely important observation emerges from the data presented in Table 1, which is the fact that, unlike unvaccinated participants, there were no statistically significant differences among vaccinated in terms of the presence or absence of DM in terms of hospitalization duration (*p* = 0.169), COVID-19 severity (*p* = 0.856), ICU admission (*p* = 0.179), and mortality (*p* = 0.179).

In Supplementary Table S1, we compared the inflammatory biomarkers according to vaccination status and the presence or absence of DM. Statistically significant differences were found between unvaccinated and vaccinated participants for certain markers, ferritin (*p* = 0.014), ESR (*p* < 0.001), LDH (*p* < 0.001) and PLR (*p* = 0.042), with higher values recorded among unvaccinated subjects. Moreover, among unvaccinated subjects, significantly higher values were recorded in DM (+) patients compared to DM (−) subjects in terms of ESR, CRP, fibrinogen, LDH, NLR and SII (*p* < 0.001), as well as for PLR (*p* = 0.036). In vaccinated subjects, however, no statistically significant differences were recorded in the values of inflammatory biomarkers according to the presence or absence of DM.

**Figure 1:**
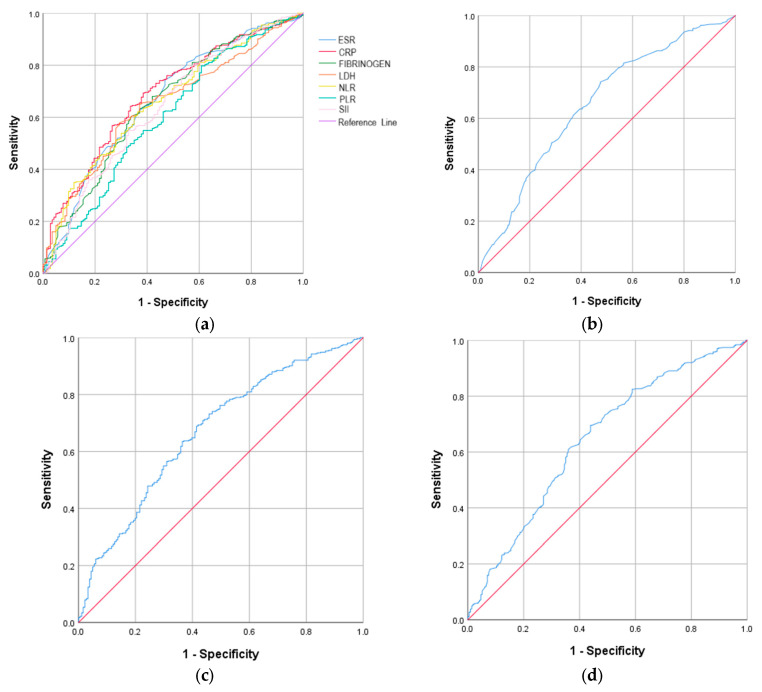

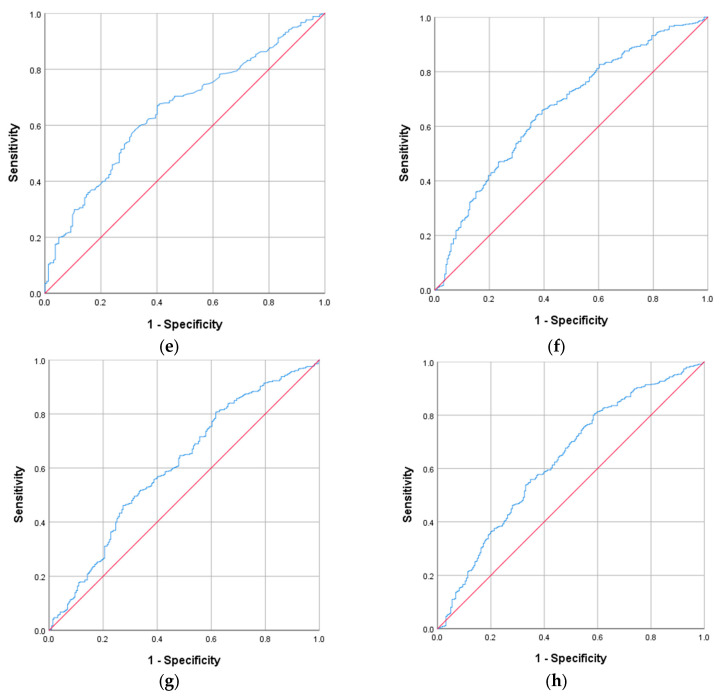
The ROC curve analysis of the inflammatory biomarkers that were significantly associated with severe COVID-19 in the unvaccinated group. (**a**) All inflammatory biomarkers; (**b**) ESR; (**c**) CRP; (**d**) fibrinogen; (**e**) LDH; (**f**) NLR; (**g**) PLR; (**h**) SII. Red line—Reference line; Blue line—ROC curve.

The discriminatory performance of inflammatory biomarkers for severe COVID-19 was evaluated using ROC analysis. Among unvaccinated subjects, most biomarkers showed comparable AUROC values, with the exception of ferritin. The highest AUROC curve was recorded for CRP, with a value of 0.668, a cut-off value of 72.30, with 57.7% sensitivity and 71.0% specificity (*p* < 0.001), followed by NLR, with a value of 0.667, the cut-off value 4.82, with 63.4% sensitivity and 64.0% specificity (*p* < 0.001) (Table 2 and Figure 1).

**Figure 2:**
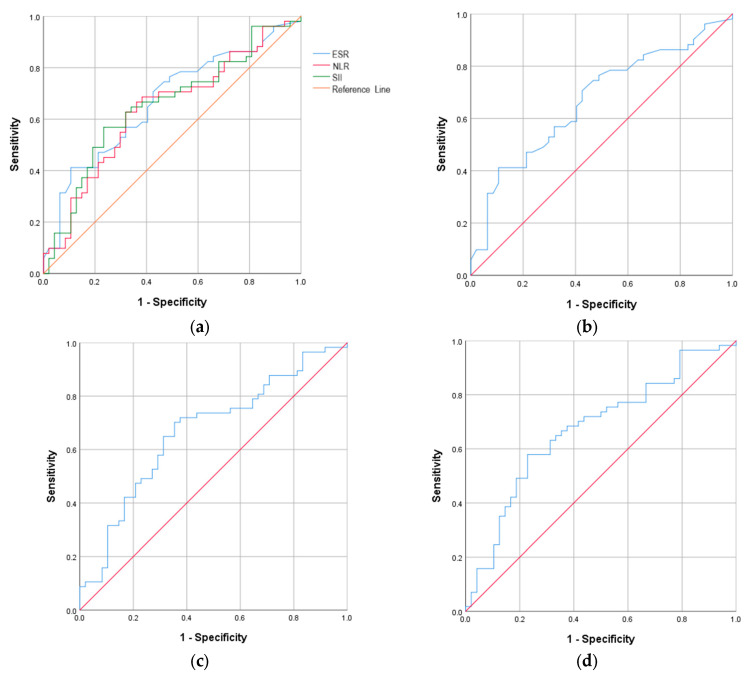
The ROC curve analysis of the inflammatory biomarkers that were significantly associated with severe COVID-19 in the vaccinated group. (**a**) All inflammatory biomarkers; (**b**) ESR; (**c**) NLR; (**d**) SII. Red line—Reference line; Blue line—ROC curve.

In the vaccinated group, the ROC curve analysis in severe COVID-19 revealed similar AUROC curves for SII, with a value of 0.694, a cut-off value of 1036.48, with 64.3% sensitivity and 71.4% specificity (*p* = 0.021); for ESR, with a value of 0.692, a cut-off value of 35.50, with 75.0% sensitivity and 61.9% specificity (*p* = 0.022); and for NLR, with a value of 0.680, a cut-off value of 4.29, with 67.9% sensitivity and 66.7% specificity (*p* = 0.032) (Table 3 and Figure 2). Statistical significance was not reached for the other analyzed inflammatory biomarkers.

An age- and comorbidities-adjusted logistic regression model was used to explore factors associated with severe COVID-19. In unvaccinated subjects, diabetes mellitus was associated with higher odds of severe disease (OR = 1.456, 95% CI 1.001–2.118, *p* = 0.049), whereas this association did not reach statistical significance in the overall cohort (OR = 1.319, 95% CI 0.940–1.851, *p* = 0.109) (Table 4). Estimates for several other covariates showed wide confidence intervals, reflecting the limited number of events and potential model instability. Multivariable analysis could not be performed in vaccinated patients because of the very small number of severe outcomes in this subgroup.

For ICU admission, multivariable analysis was conducted using a parsimonious model including age and major comorbidities. Diabetes mellitus showed an association with increased odds of ICU admission both in the unvaccinated group (OR = 3.649, 95% CI 1.782–7.473, *p* < 0.001) and in the overall cohort (OR = 4.076, 95% CI 2.005–8.283, *p* < 0.001) (Table 5). For some less frequent comorbidities, reliable estimates could not be obtained because no ICU events occurred in those categories, underscoring the limitations posed by sparse data. In vaccinated patients, modeling was not feasible, as only one vaccinated subject with diabetes required ICU care.

In the mortality analysis, diabetes mellitus was associated with higher odds of death both in unvaccinated subjects (OR = 33.668, 95% CI 4.510–251.340, *p* = 0.001) and in the overall cohort (OR = 37.103, 95% CI 4.999–275.410, *p* < 0.001) (Table 6). The very wide confidence intervals indicate limited precision and substantial uncertainty of these estimates, most likely related to the small number of mortality events. Results from this model should therefore be interpreted as exploratory. Multivariable analysis could not be performed in vaccinated patients because only one death occurred in this subgroup.

Overall, the magnitude of several odds ratios should be interpreted cautiously, as sparse events limited model stability despite the use of a parsimonious set of clinically relevant covariates.

## 3. Discussion

DM as a risk factor for adverse COVID-19 outcomes has been the focus of substantial attention [13,34,35,36,37]. A systematic review and meta-analysis undertaken by Fatoke et al. [38] confirms a significant association between the presence of DM and increased risk of mortality in patients with COVID-19, as well as a significant association with more severe COVID-19 disease and the requirement for ventilatory support, results that are consistent with those reported in other studies [35,39,40,41]. Our results are in line with this literature; however, in contrast to some previous reports, the association between DM and severe COVID-19 was observed primarily in unvaccinated subjects, but it did not reach statistical significance in the overall cohort. Importantly, estimates from our mortality model were affected by wide confidence intervals, indicating limited precision and the need for cautious interpretation. Differences across geographic regions, as highlighted by Kastora et al. [13], may further explain variability between studies. Accordingly, in Europe and America, no statistically significant differences were reported in terms of mortality, ICU admission, and COVID-19 severity among subjects with DM, possibly due to an extremely small number of studies included in the meta-analysis [13].

Furthermore, accumulating evidence points to potential differences in viral replication, load, and persistence between patients with and without DM. Hyperglycemia may favor prolonged viral shedding and elevated viral burden [38,40]. The association between the two conditions arises from the intricate pathophysiological processes underlying both DM and COVID-19, leading to amplified immune responses, intensified inflammatory states, and impaired pulmonary and cardiovascular function [38,42,43].

Regarding inflammatory biomarkers, multiple variables assessed in previously published research have shown associations with COVID-19 severity [18,21,23,24]. In 2025, Kumar et al. [44] published a study in which elevated CRP, ESR, LDH and ferritin values were found in patients with severe COVID-19, demonstrating a significant role in predicting disease severity. Similar results were described in our study in unvaccinated patients, except for ferritin, which did not reach statistical significance. NLR has also been associated with an important role in predicting COVID-19 severity [44], results that are consistent with those reported in our study. Another study published in 2020 by Yang et al. [45] reported AUROC values for NLR, CRP and PLR, suggesting their use as supportive risk indicators in severe COVID-19 rather than as standalone diagnostic tools. The results regarding PLR are also consistent with the results reported by our team in unvaccinated subjects. Sakthivadivel et al. [18] reported that CRP and NLR are associated with worse outcomes and may contribute to early risk stratification when interpreted alongside clinical assessment. Furthermore, the study published by Liao et al. [46] in 2020 revealed that increased NLR served as a valuable predictor of COVID-19 severity among ICU-admitted patients. Numerous other studies have highlighted the importance of elevated CRP as a predictive biomarker for COVID-19 severity, ICU admission or mortality [18,47,48,49,50]. A high SII value was likewise found to be associated with severe COVID-19 in a study conducted by Muhammad et al. in 2021 [51], results that are in agreement with our study’s findings in both vaccinated and unvaccinated subjects. Additional studies have documented markedly increased SII levels among individuals with severe COVID-19 [52], emphasizing the biomarker’s utility in severity assessment and its association with delayed negative nucleic acid conversion [53]. In our cohort, the discriminatory performance of these biomarkers was moderate (AUROC ≈ 0.66–0.69), indicating that they should be viewed as complementary indicators rather than independent clinical prediction tools. Overall, these studies [18,54], together with our findings, support the potential utility of inflammatory biomarkers for risk stratification in COVID-19, although their predictive value may vary according to vaccination status and clinical context.

All these data highlight the need for focused interventions, emphasizing optimized glycemic control, individualized management of comorbidities, and potential anti-inflammatory interventions in order to improve prognosis in this susceptible population [38,41]. They also open new directions for research, particularly concerning the relationship between DM, immune responses, and cardiovascular involvement in viral infections [38].

It is critically important that vaccination has been, and remains, the most effective strategy for reducing COVID-19 severity and mortality [9]. In a study carried out in our country by Briciu et al. [9], it was shown that unvaccinated patients were older, had a higher prevalence of cardiovascular comorbidities, experienced prolonged hospitalization, and exhibited higher rates of severe/critical COVID-19, ICU admission, and mortality. These observations are largely consistent with our findings, except for age and associated cardiovascular diseases, as both unvaccinated and vaccinated groups demonstrated similar distributions for these variables. The discrepancies between our study and that of Briciu et al. should be interpreted in the context of the distinct enrollment periods, which corresponded to different pandemic phases with evolving variants and standards of care rather than to a direct causal effect of vaccination. Another study conducted in our country by Manole et al. [55] identified increased ICU admission and mortality rates in unvaccinated subjects, supporting the overall contextual pattern observed in our analysis. Laitin et al. [56] reported a longer mean hospitalization period in unvaccinated versus vaccinated COVID-19 patients, but without statistical significance. These results diverge from our findings, which showed a significant difference (11 vs. 6 days, *p* < 0.001). The 2022 analysis by Fatima et al. [57] likewise reported increased rates of severe/critical COVID-19, ICU admission, and mechanical ventilation among unvaccinated individuals compared with those who were vaccinated. It should be emphasized, however, that comparisons between vaccinated and unvaccinated groups in observational cohorts reflect real-world epidemiological contexts and may be influenced by temporal and clinical heterogeneity. It is essential to note that although vaccines may not offer absolute protection against infection, they remain highly effective in mitigating disease severity.

However, our study has limitations that need to be considered. An important limitation relates to the temporal structure of the cohort. Because vaccination status and calendar period were highly overlapping variables, adjustment for the admission period within multivariable models would have introduced substantial collinearity and unstable estimates. For this reason, analyses were interpreted as comparisons between two real-world pandemic contexts rather than as strictly causal effects of vaccination. Nevertheless, this timeframe allowed for the assessment of disease severity and inflammatory response according to vaccination status and the presence of diabetes mellitus under different viral and public-health conditions.

Furthermore, the study was conducted in a specific geographical region; therefore, the results cannot be extrapolated to the general population and can only be applied to limited populations with similar characteristics. Therefore, we must take into account inter-ethnic or racial differences, demographic factors, environmental factors, lifestyle patterns and accessibility to public healthcare, all of which may influence the risk of COVID-19 unfavorable outcomes, as well as associated comorbidities. Another aspect that must be taken into consideration when interpreting our results is the study’s retrospective, observational design, which does not allow for the establishment of a cause–effect relationship between inflammatory biomarkers and severe forms of COVID-19. Additional data can be provided by an analysis of evolutionary changes in these biomarkers. Moreover, in the logistic regression analysis, a small number of subjects were assigned to some subgroups, thus possibly leading to the wide confidence intervals. Some multivariable estimates, particularly for mortality, were based on a limited number of events, resulting in wide confidence intervals and potential model instability; these findings should be regarded as exploratory until validated in larger cohorts using penalized regression approaches. A further limitation relates to the assessment of vaccination status: details were partly obtained from patient self-report, and information on vaccine type, dosing schedule, and interval since the last dose was incomplete, potentially leading to misclassification and heterogeneity within the vaccinated cohort. Aditionally, DM was primarily analyzed as a binary variable; although information on antidiabetic treatment was available, data on HbA1c and disease duration were not systematically recorded. Given the known influence of long-term glycemic control on COVID-19 outcomes, the observed associations should be interpreted with caution.

This study also presents strengths. One of these is represented by the sample size, with a large number of subjects included in the study. Moreover, biochemical data were obtained using standardized measurement procedures. Another strength lies in the fact that the diagnosis of COVID-19 was determined based on reverse transcription–polymerase chain reaction (RT-PCR) or antigen assays. To date, to our knowledge, this is the first study in our country that analyzes data according to vaccination status and DM diagnosis.

To date, the subject we investigated in this study remains insufficiently explored in our country. Replication across multiethnic, multicenter populations is essential to improve relevance and ensure result validation. Longitudinal studies are required to clarify the causal link between COVID-19 and inflammatory biomarkers and DM, respectively, as well as vaccination influence among patients with associated comorbidities, in order to enhance clinical practice and contribute to prevention.

## 4. Materials and Methods

### 4.1. Study Design

We performed a retrospective, observational study, conducted between October 2020 and January 2021 and between January and December 2022, using the Strengthening the Reporting of Observational Studies in Epidemiology (STROBE) Statement [58]. The study period was deliberately defined to include two epidemiologically distinct stages of the COVID-19 pandemic in Romania. The first interval (October 2020–January 2021) preceded the large-scale implementation of vaccination and corresponded to the circulation of early SARS-CoV-2 variants, providing a representative cohort of predominantly unvaccinated patients. The second interval (January–December 2022) reflected the post-vaccination era, largely dominated by Delta and Omicron variants, when vaccination coverage had increased substantially. This timeframe allowed a comparative assessment of disease severity and inflammatory response according to vaccination status and the presence of diabetes mellitus, under different viral and public-health conditions. The sample size was determined using calculator.net [59]. The minimum required sample size was estimated at approximately 405 participants. However, the final cohort included 866 subjects in order to allow subgroup comparisons according to vaccination status and diabetes mellitus and to ensure adequate stability of multivariable logistic regression models for severe COVID-19, ICU admission, and mortality. Given the expected imbalance between groups and the need for sufficient outcome events per predictor variable, the expanded cohort was considered necessary to provide reliable and representative epidemiological analyses.

### 4.2. Population

The study included participants who met the following criteria: age over 18 years, subjects confirmed positive for SARS-CoV-2 by RT-PCR or antigen tests, and those who underwent evaluation for the associated comorbidities and vaccination status, during hospitalization at the Institute of Pneumophtisiology “Marius Nasta” Bucharest, a national referral center for chronic pulmonary diseases. Exclusion criteria comprised: age under 18 years, subjects who tested negative for SARS-CoV-2 by RT-PCR or antigen assays, and other periods except those mentioned in the data collection method. All participants were enrolled in the study after signing the informed consent form. The study was performed in accordance with the World Medical Association Declaration of Helsinki—Ethical Principles for Medical Research Involving Human Participants, as well as with the applicable standards of the International Conference on Harmonization (ICH)/Good Clinical Practice (GCP), and was approved by the Scientific Ethics Committee of the Institute of Pneumophtisiology “Marius Nasta” Bucharest, approval number 4913/J 3 March 2023.

### 4.3. Data Source

Relevant socio-demographic characteristics (gender, age, environment) were recorded, as well as the medical history of associated comorbidities (DM, cardiovascular diseases, obesity, neurological, endocrine, hepatic, renal, pulmonary, rheumatological, psychiatric, hematological diseases, cancer and HIV) and vaccination status: unvaccinated, incomplete vaccination (one of two primary doses), complete primary vaccination, and booster vaccination. Vaccination status was recorded at hospital admission based on medical documentation and patient interview. Patients were classified as vaccinated if they reported receiving at least one dose of any authorized SARS-CoV-2 vaccine prior to admission. The number of doses was obtained from patient declarations, as systematic linkage with the national vaccination registry was not feasible for all cases. Information regarding vaccine platform, homologous or heterologous schedules, and the exact interval between the last dose and symptom onset was not consistently available in the medical records; therefore, these variables could not be incorporated into the analyses.

### 4.4. Clinical and Biochemical Data

Clinical investigators classified the comorbidities based on documented diagnoses in the electronic patient file, using the International Statistical Classification of Diseases and Related Health Problems 10th Revision [60].

Biochemical data were obtained from venous blood collected in a vacutainer, labeled with each participant’s identification number. Analyses were performed in accordance with standardized laboratory procedures. All analyzed blood samples were obtained at hospital admission (baseline), prior to initiation of specific in-hospital therapy. Fasting plasma glucose (FPG) and lactate dehydrogenase (LDH) were determined through enzymatic methods. Ferritin and C-reactive protein (CRP) were analyzed using an immunoassay. Fibrinogen measurements were performed by turbidimetry, whereas erythrocyte sedimentation rate (ESR) was determined using the Westergren method. The complete blood count was obtained using the laser flow cytometry method. Additionally, specific inflammatory biomarkers were calculated according to the formulas presented in Table 7.

### 4.5. COVID-19 Evaluation

The severity of COVID-19 was classified as asymptomatic, mild (without pneumonia), moderate (non-severe pneumonia) and severe/critical (severe: tachypnea ≥ 30 breaths/min, oxygen saturation (SpO_2_) ≤ 93%, arterial partial pressure of oxygen and the fraction of inspired oxygen ratio (PaO_2_/FIO_2_) < 300 mmHg and/or lung involvement exceeding 50% of the pulmonary field within 24–48 h; critical: respiratory failure requiring ventilatory support, septic shock and/or multiple organ failure), following the initial WHO classification [61] and its adoption in Romania through a Health Ministry Order on COVID-19 management [9]. Disease severity was assessed at the end of hospitalization. The length of hospitalization, ICU admission and clinical endpoints, either mortality or discharge, were registered.

### 4.6. Comorbidities Evaluation

The comorbidities evaluated in this study were selected on the basis of published evidence identifying them as major predictors of adverse COVID-19 outcomes, including disease severity, need for intensive care, and mortality [13,14,15,34,35,36,37,38,39,40,41,62]. Cardiovascular diseases, diabetes mellitus, obesity, chronic pulmonary, renal, hepatic, neurological, endocrine, rheumatological, psychiatric, hematological disorders, cancer, and HIV infection have been consistently reported as conditions that worsen the clinical course of SARS-CoV-2 infection. These diagnoses were retrieved from the hospital electronic records and coded according to the International Classification of Diseases, 10th Revision (ICD-10) [60], ensuring a standardized and reproducible case definition across participants. In line with the STROBE Statement for observational research [58], the selected comorbidities were further used as covariates in the multivariable logistic regression models in order to control for potential confounding and to provide adjusted estimates of the association between diabetes, vaccination status, and COVID-19 outcomes.

### 4.7. Assessment of Diabetes Mellitus

DM was defined based on documented medical history or ongoing antidiabetic therapy at the time of admission. When available, information on diabetes management—including outpatient treatment with oral antidiabetic agents, non-insulin injectable therapy, chronic insulin therapy, and the need for insulin administration during hospitalization—was extracted from medical records. HbA1c values and duration of diabetes were not systematically available, as patients were admitted to a Pneumology hospital where prior metabolic control parameters were not routinely documented.

### 4.8. Statistical Analysis

The distribution of continuous variables was assessed using the Kolmogorov–Smirnov test, which indicated a non-Gaussian pattern. Accordingly, variables were reported as median and interquartile range (IQR). Differences between groups were assessed using the Mann–Whitney U test. Categorical variables were compared using the chi-square test. Cut-off values for inflammatory biomarkers were established by evaluating the area under the receiver operating characteristic (AUROC) curve. Because vaccination status and calendar period were strongly collinear in our dataset, adjustment for admission period was not included in the multivariable models to avoid unstable estimates. Statistical significance was defined as *p* < 0.05. Data processing was performed with the Statistical Package for the Social Sciences (SPSS) version 26.0 (SPSS Inc., Chicago, IL, USA).

## 5. Conclusions

In unvaccinated subjects, we observed a significantly higher frequency of severe COVID-19, ICU admission and mortality. Also, the duration of hospitalization was almost double compared to vaccinated participants. In unvaccinated DM (+) patients, compared to unvaccinated DM (−) patients, a longer duration of hospitalization, a higher percentage of moderate and severe COVID-19, a higher frequency of ICU admission, and an alarming frequency of mortality were reported. Among vaccinated subjects, these variables did not show statistically significant differences according to the presence or absence of DM, supporting the interpretation that vaccination attenuates the excess risk associated with diabetes.

Regarding inflammatory biomarkers, in unvaccinated patients, the highest discriminatory performance for severe COVID-19 was recorded for CRP, whereas in vaccinated participants, SII presented the highest AUROC. These biomarkers demonstrated moderate discrimination and should be regarded as complementary indicators that support, but do not replace, clinical assessment. In unvaccinated subjects, their age- and other comorbidities-adjusted model for multivariate logistic regression analysis demonstrated that DM was associated with higher odds of severe COVID-19, intensive care need and mortality; nevertheless, these estimates were affected by wide confidence intervals and should be interpreted cautiously. Within the vaccinated group, the multivariable analysis could not be performed due to the extremely low number of subjects who presented unfavorable outcomes.

The main contributions of this study are: (1) the demonstration, in a real-world Romanian cohort, of the attenuation of diabetes-related risk within the post-vaccination pandemic context; (2) the identification of readily available inflammatory indices—particularly CRP and SII—as useful tools for early risk stratification according to vaccination status; and (3) the comprehensive evaluation of multiple comorbidities with adjusted models, providing a nuanced framework for clinical decision-making.

Our findings emphasize the crucial role of vaccination in reducing comorbidity-related risk, most notably that conferred by DM, and support the development of individualized strategies for the timely recognition and management of patients with a high likelihood of adverse outcomes. These results should be interpreted within the specific epidemiological phases of the pandemic, acknowledging the temporal differences between cohorts and the contextual nature of the comparisons.

## Figures and Tables

**Table 1:** Distribution of variables in the study population.

Variables	Total (n = 866)			Unvaccinated (n = 760)			Vaccinated (n = 106)		
	Unvaccinated	Vaccinated	*p* Value	DM (+) (n = 488)	DM (−) (n = 272)	*p* Value	DM (+) (n = 38)	DM (−) (n = 68)	*p* Value
Age (years)	68 (16)	69 (19)	0.504	67 (13)	68 (20)	0.05	70 (12)	67 (25)	0.204
Age ≥ 65 years (%)	61.3	59.4	0.710	60.9	62.1	0.730	71.1	52.9	0.069
Males (%)	56.4	55.7	0.887	57.7	54.0	0.330	60.5	52.9	0.451
Urban environment (%)	74.9	86.8	0.007	77.3	70.6	0.042	86.8	86.8	0.991
Vaccine doses (%)									
0/1	100.0	9.4	<0.001	100.0	100.0	-	18.4	4.4	0.030
2	0.0	51.9		0.0	0.0		39.5	58.8	
3	0.0	38.7		0.0	0.0		42.1	36.8	
FPG (mg/dL)	145.0 (99.0)	110.5 (58.0)	<0.001	175.0 (118.0)	111.0 (51.0)	<0.001	158.0 (128.0)	105.0 (24.0)	<0.001
Comorbidities (%)									
DM	64.2	35.8	<0.001	100.0	0.0	-	100.0	0.0	-
Cardiovascular	84.3	79.2	0.183	88.5	76.8	<0.001	94.7	70.6	0.003
Obesity	28.7	22.6	0.194	34.8	17.6	<0.001	34.2	16.2	0.033
Neurological	7.1	12.3	0.063	4.1	12.5	<0.001	10.5	13.2	0.683
Endocrine	5.5	6.6	0.653	5.3	5.9	0.748	5.3	7.4	0.678
Hepatic	26.4	17.0	0.036	31.1	18.0	<0.001	31.6	8.8	0.003
Renal	25.8	17.0	0.049	31.1	16.2	<0.001	28.9	10.3	0.014
Pulmonary	20.1	27.4	0.087	11.7	35.3	<0.001	26.3	27.9	0.857
Rheumatological	3.3	9.4	0.003	1.4	6.6	<0.001	7.9	10.3	0.685
Psychiatric	10.4	6.6	0.220	9.4	12.1	0.245	2.6	8.8	0.218
Hematological	47.9	49.1	0.823	54.3	36.4	<0.001	60.5	42.6	0.077
Cancer	8.0	17.9	0.001	5.7	12.1	0.002	21.1	16.2	0.530
HIV	0.7	1.9	0.186	0.0	1.8	0.003	0.0	2.9	0.286
Outpatient DM treatment									
Oral medication	31.4	23.6	0.103	48.6	0.0	<0.001	65.8	0.0	<0.001
Non-insulin injectable medication	2.0	0.0	0.144	3.1	0.0	0.004	0.0	0.0	–
Insulin	12.4	11.3	0.754	19.3	0.0	<0.001	31.6	0.0	<0.001
Insulin therapy during hospitalization	55.3	16.0	<0.001	83.6	0.0	<0.001	39.5	0.0	<0.001
Hospitalization duration (days)	11 (7)	6 (5)	<0.001	12 (8)	8 (6)	<0.001	8 (6)	6 (5)	0.169
Disease severity (%)									
Asymptomatic	0.1	0.9	<0.001	0.0	0.4	<0.001	0.0	1.5	0.856
Mild	11.3	25.5		4.1	24.3		26.3	25.0	
Moderate	17.9	18.9		20.1	14.0		21.1	17.6	
Severe/critical	70.7	54.7		75.8	61.4		52.6	55.9	
ICU admission (%)	12.1	0.9	0.001	16.8	3.7	<0.001	2.6	0.0	0.179
Mortality (%)	9.5	0.9	0.003	14.5	0.4	<0.001	2.6	0.0	0.179

DM: diabetes mellitus; 0: unvaccinated, 1: incomplete vaccination; 2: complete primary vaccination; 3: booster vaccination; FPG: fasting plasma glucose; HIV: human immunodeficiency virus; ICU: intensive care unit. Continuous variables with abnormal distribution are presented as median (IQR), and those with normal distribution are presented as mean ± standard deviation.

**Table 2:** The ROC curve analysis for the inflammatory biomarkers in the unvaccinated group in severe COVID-19.

Inflammatory Biomarkers	AUROC Curve	Standard Error	95% CI	*p* Value	Cut-Off Value	Sensitivity (%)	Specificity (%)
Ferritin	0.547	0.034	0.480–0.615	0.162	–	–	–
ESR	0.659	0.033	0.594–0.724	<0.001	60.50	66.0	61.0
CRP	0.668	0.031	0.608–0.728	<0.001	72.30	57.7	71.0
Fibrinogen	0.644	0.032	0.581–0.707	<0.001	445.50	60.0	63.0
LDH	0.634	0.031	0.573–0.695	<0.001	279.50	57.7	71.0
NLR	0.667	0.031	0.606–0.728	<0.001	4.82	63.4	64.0
PLR	0.590	0.034	0.523–0.657	0.008	240.43	55.5	61.0
SII	0.645	0.032	0.582–0.709	<0.001	1292.80	55.8	68.0

ROC: receiver operating characteristic; AUROC: area under the receiver operating characteristic; CI: confidence interval; ESR: erythrocyte sedimentation rate; CRP: C-reactive protein; LDH: lactate dehydrogenase; NLR: neutrophil-to-lymphocyte ratio; PLR: platelet-to-lymphocyte ratio; SII: systemic immune-inflammation index.

**Table 3:** The ROC curve analysis for the inflammatory biomarkers in the vaccinated group in severe COVID-19.

Inflammatory Biomarkers	AUROC Curve	Standard Error	95% CI	*p* Value	Cut-Off Value	Sensitivity (%)	Specificity (%)
Ferritin	0.583	0.084	0.418–0.749	0.322	–	–	–
ESR	0.692	0.079	0.537–0.847	0.022	35.50	75.0	61.9
CRP	0.641	0.084	0.477–0.806	0.094	–	–	–
Fibrinogen	0.636	0.086	0.467–0.805	0.106	–	–	–
LDH	0.633	0.080	0.475–0.790	0.115	–	–	–
NLR	0.680	0.077	0.530–0.830	0.032	4.29	67.9	66.7
PLR	0.641	0.081	0.482–0.801	0.094	–	–	–
SII	0.694	0.076	0.545–0.842	0.021	1036.48	64.3	71.4

ROC: receiver operating characteristic; AUROC: area under the receiver operating characteristic; CI: confidence interval; ESR: erythrocyte sedimentation rate; CRP: C-reactive protein; LDH: lactate dehydrogenase; NLR: neutrophil-to-lymphocyte ratio; PLR: platelet-to-lymphocyte ratio; SII: systemic immune-inflammation index.

**Table 4:** The multivariate logistic regression analysis model predicting severe COVID-19, adjusted for age and comorbidities.

Variables	Total			Unvaccinated		
	OR	95% CI	*p* Value	OR	95% CI	*p* Value
DM	1.319	0.940–1.851	0.109	1.456	1.001–2.118	0.049
Age ≥ 65 years	1.299	0.923–1.829	0.133	1.342	0.922–1.953	0.124
Cardiovascular	2.371	1.565–3.592	<0.001	2.525	1.605–3.973	<0.001
Obesity	1.624	1.119–2.356	0.011	1.475	0.988–2.202	0.057
Neurological	0.590	0.345–1.010	0.055	0.521	0.287–0.945	0.032
Endocrine	0.644	0.341–1.213	0.173	0.534	0.269–1.058	0.072
Hepatic	1.174	0.815–1.692	0.389	1.248	0.842–1.850	0.270
Renal	1.206	0.818–1.780	0.344	1.171	0.770–1.780	0.460
Pulmonary	0.994	0.680–1.453	0.976	1.146	0.743–1.768	0.537
Rheumatological	0.782	0.375–1.634	0.513	1.012	0.408–2.509	0.980
Psychiatric	1.179	0.688–2.021	0.548	1.132	0.642–1.997	0.668
Hematological	1.219	0.890–1.669	0.217	1.200	0.851–1.690	0.298
Cancer	0.653	0.396–1.075	0.094	0.575	0.325–1.017	0.057
HIV	0.771	0.158–3.757	0.747	0.522	0.079–3.450	0.500

OR: odds ratio; CI: confidence interval; DM: diabetes mellitus; HIV: human immunodeficiency virus.

**Table 5:** The multivariate logistic regression analysis model predicting ICU admission need, adjusted for age and comorbidities.

Variables	Total			Unvaccinated		
	OR	95% CI	*p* Value	OR	95% CI	*p* Value
DM	4.076	2.005–8.283	<0.001	3.649	1.782–7.473	<0.001
Age ≥ 65 years	1.181	0.709–1.966	0.523	1.185	0.707–1.985	0.519
Cardiovascular	2.733	0.946–7.894	0.063	2.862	0.990–8.280	0.052
Obesity	1.974	1.234–3.160	0.005	1.955	1.212–3.155	0.006
Neurological	0.876	0.294–2.612	0.812	0.969	0.320–2.939	0.956
Endocrine	0.258	0.059–1.124	0.071	0.246	0.056–1.081	0.063
Hepatic	1.031	0.625–1.700	0.905	0.992	0.596–1.652	0.976
Renal	1.924	1.189–3.114	0.008	1.877	1.151–3.061	0.012
Pulmonary	1.175	0.629–2.196	0.613	1.184	0.619–2.262	0.610
Rheumatological *	0.000	–	0.998	0.000	–	0.998
Psychiatric	0.786	0.353–1.749	0.555	0.741	0.332–1.652	0.463
Hematological	1.204	0.752–1.927	0.440	1.197	0.745–1.924	0.457
Cancer	0.742	0.277–1.984	0.552	0.924	0.341–2.500	0.876
HIV *	0.000	–	0.999	0.000	–	0.999

ICU: intensive care unit; OR: odds ratio; CI: confidence interval; DM: diabetes mellitus; HIV: human immunodeficiency virus. * 95% CI could not be calculated.

**Table 6:** The multivariate logistic regression analysis model predicting mortality, adjusted for age and other comorbidities.

Variables	Total			Unvaccinated		
	OR	95% CI	*p* Value	OR	95% CI	*p* Value
DM	37.103	4.999–275.410	<0.001	33.668	4.510–251.340	0.001
Age ≥ 65 years	1.622	0.860–3.061	0.135	1.614	0.850–3.064	0.143
Cardiovascular	6.041	0.794–45.985	0.082	6.457	0.848–49.166	0.072
Obesity	1.117	0.641–1.946	0.697	1.048	0.594–1.850	0.871
Neurological	1.444	0.443–4.713	0.543	1.620	0.482–5.452	0.436
Endocrine	0.450	0.123–1.650	0.229	0.424	0.115–1.563	0.197
Hepatic	1.062	0.595–1.895	0.840	1.015	0.561–1.836	0.961
Renal	4.439	2.563–7.688	<0.001	4.384	2.512–7.651	<0.001
Pulmonary	0.628	0.251–1.572	0.321	0.592	0.219–1.595	0.300
Rheumatological *	0.000	–	0.997	0.000	–	0.998
Psychiatric	1.411	0.632–3.148	0.400	1.319	0.588–2.959	0.502
Hematological	1.505	0.857–2.643	0.155	1.478	0.836–2.612	0.179
Cancer	0.834	0.268–2.596	0.754	0.982	0.307–3.137	0.976
HIV *	0.000	–	0.999	0.000	–	0.999

OR: odds ratio; CI: confidence interval; DM: diabetes mellitus; HIV: human immunodeficiency virus. * 95% CI could not be calculated.

**Table 7:** Inflammatory biomarkers definition.

Biomarker	Formula	Reference
NLR	neutrophil count (×10^3^ cells/μL)/lymphocyte count (×10^3^ cells/μL)	[45]
PLR	platelet count (×10^3^ cells/μL)/lymphocyte count (×10^3^ cells/μL)	[45]
SII	platelet (×10^3^ cells/μL) × (neutrophil count (×10^3^ cells/μL)/lymphocyte count (×10^3^ cells/μL))	[53]

NLR: neutrophil-to-lymphocyte ratio; PLR: platelet-to-lymphocyte ratio; SII: systemic immune-inflammation index.

## Data Availability

The data supporting the findings of this study are available within the article and its Supplementary Material. Additional anonymized data may be available from the corresponding author upon reasonable request and with permission of the Ethics Committee of the Institute of Pneumophtisiology “Marius Nasta” Bucharest.

## Supplementary Materials

The following supporting information can be downloaded at: https://www.mdpi.com/article/10.3390/ijms27042082/s1.

## Abbreviations

The following abbreviations are used in this manuscript:

AUROC	area under the receiver operating characteristic
COVID-19	coronavirus disease 2019
CI	confidence interval
CRP	C-reactive protein
DM	diabetes mellitus
ESR	erythrocyte sedimentation rate
FIO_2_	fraction of inspired oxygen
FPG	fasting plasma glucose
GCP	Good Clinical Practice
HIV	human immunodeficiency virus
ICH	International Conference on Harmonization
ICU	intensive care unit
IL-2	interleukin-2
IL-6	interleukin-6
IL-7	interleukin-7
IL-10	interleukin-10
IQR	interquartile range
LDH	lactate dehydrogenase
MODS	multiple organ dysfunction syndrome
NLR	neutrophil-to-lymphocyte ratio
OR	odds ratio
PaO_2_	arterial partial pressure of oxygen
PLR	platelet-to-lymphocyte ratio
ROC	receiver operating characteristic
RT-PCR	reverse transcription–polymerase chain reaction
SARS-CoV-2	severe acute respiratory syndrome coronavirus 2
SII	systemic immune-inflammation index
SpO_2_	oxygen saturation
SPSS	Statistical Package for the Social Sciences
STROBE	The Strengthening the Reporting of Observational Studies in Epidemiology
TNF	tumor necrosis factor
VOC	variant of concern
WHO	World Health Organization
