# Supplementary material for: Comparison of Severe COVID-19 Outcomes in Vaccinated and Unvaccinated Patients, with and Without Diabetes Mellitus in a Romanian Tertiary Healthcare Pneumology Hospital—A Retrospective Study
Source: Int J Mol Sci. 2026 Feb 23;27(4):2082. doi: 10.3390/ijms27042082 (PMC12940379; doi:10.3390/ijms27042082)
Supplement: Supplementary file 1 [file ijms-27-02082-s001.zip › ijms-4067422-supplementary.pdf]

Supplementary Table S1. Inflammatory biomarkers according to vaccination status and DM.

Inflammatory biomarkers	Total			Unvaccinated			Vaccinated		
	**Unvaccinated**	**Vaccinated**	**p value**	**DM (+)**	**DM (−)**	**p value**	**DM (+)**	**DM (−)**	**p value**
Ferritin	542.00 (411.50)	379.20 (449.15)	0.014	558.00 (368.25)	430.00 (705.20)	0.136	473.60 (427.40)	310.20 (442.10)	0.272
ESR	71.00 (57.00)	45.00 (55.00)	<0.001	80.00 (50.00)	30.00 (43.00)	<0.001	60.00 (70.00)	40.50 (56.00)	0.321
CRP	72.12 (113.83)	50.73 (115.96)	0.111	81.95 (118.34)	33.86 (93.67)	<0.001	63.38 (161.72)	45.16 (108.96)	0.507
Fibrinogen	457.00 (144.00)	414.00 (187.00)	0.056	471.50 (138.00)	388.00 (162.00)	<0.001	417.00 (201.00)	403.50 (169.00)	0.814
LDH	279.00 (192.00)	220.00 (198.00)	<0.001	294.00 (195.00)	236.00 (143.00)	<0.001	227.00 (279.00)	205.50 (124.00)	0.374
NLR	5.44 (6.12)	4.65 (4.96)	0.153	5.69 (6.63)	4.89 (5.91)	<0.001	5.85 (3.76)	3.82 (7.57)	0.163
PLR	244.34 (227.45)	198.96 (169.81)	0.042	258.84 (229.50)	205.33 (197.52)	0.036	251.53 (135.05)	192.28 (184.44)	0.091
SII	1234.07 (2140.90)	1027.45 (1709.95)	0.375	1338.21 (2332.04)	956.85 (1764.76)	<0.001	1157.82 (1678.61)	999.32 (1780.41)	0.533

DM: diabetes mellitus; ESR: erythrocyte sedimentation rate; CRP: C-reactive protein; LDH: lactate dehydrogenase; NLR: neutrophil-to-lymphocyte ratio; PLR: platelet-to-lymphocyte ratio; SII: systemic immune-inflammation index. Continuous variables with abnormal distribution are presented as median (IQR) and those with normal distribution are presented as mean ± standard deviation.
